# Relationship Between Intracranial Pressure, Ocular Blood Flow and Vessel Density: Insights from OCTA and Doppler Imaging

**DOI:** 10.3390/medicina61050800

**Published:** 2025-04-25

**Authors:** Arminas Zizas, Keren Wood, Austėja Judickaitė, Vytautas Petkus, Arminas Ragauskas, Viktorija Bakstytė, Alon Harris, Ingrida Janulevičienė

**Affiliations:** 1 Department of Ophthalmology, Lithuanian University of Health Sciences, LT-44307 Kaunas, Lithuania; arminas.zizas@lsmu.lt (A.Z.); austeja.judickaite@lsmu.lt (A.J.); viktorija.bakstyte@gmail.com (V.B.); 2 Department of Ophthalmology, Icahn School of Medicine at Mount Sinai, New York, NY 10029, USA; keren.woodshalem@mssm.edu (K.W.); alon.harris@mssm.edu (A.H.); 3 Health Telematics Science Institute, Kaunas University of Technology, LT-51423 Kaunas, Lithuania; vytautas.petkus@ktu.lt (V.P.); arminas.ragauskas@ktu.lt (A.R.)

**Keywords:** glaucoma, intracranial pressure, ocular blood flow, optical coherence tomography angiography, optic nerve head, retinal nerve fiber layer, swept-source optical coherence tomography, vessel density, Doppler

## Abstract

*Background and Objectives*: Despite the growing amount of new research, the pathophysiology of glaucoma remains unclear. The aim of this study was to determine the relationship between intracranial pressure (ICP), ocular blood flow and structural optic nerve parameters. *Materials and Methods:* A prospective clinical study was conducted involving 24 patients with open-angle glaucoma and 25 healthy controls. Routine clinical examination was performed. Swept-source optical coherence tomography (SS-OCT) and OCT angiography (OCTA) images were taken (DRI-OCT Triton, Topcon). The vessel density (VD) values of the ONH were calculated around the optic nerve head (ONH). An orbital Doppler device (Vittamed 205, Kaunas, Lithuania) was used for non-invasive ICP measurements. Color Doppler imaging (CDI) (Mindray M7, Shenzhen, China) was used for retrobulbar blood flow measurements in the ophthalmic artery (OA), central retinal artery (CRA) and short posterior ciliary arteries (SPCAs). *Results*: ICP was 8.35 ± 2.8 mmHg in the glaucoma group and 8.45 ± 3.19 mmHg in the control group (*p* = 0.907). In the glaucoma group, the VD of the superficial vascular plexus in the inferior-nasal (NI) sector of the ONH showed a correlation with ICP (r = 0.451, *p* = 0.05). In contrast, the control group exhibited weaker correlations. CRA peak systolic velocity (PSV) demonstrated significant moderate correlations with VD in multiple retinal layers, including the avascular retina layer in the temporal (T) sector (r = 0.637, *p* = 0.001). *Conclusions*: Lower ICP was significantly associated with the lower VD of the superficial plexus layer in the inferior-nasal sector in the glaucoma group, with the control group exhibiting weaker correlations in all sectors. Further longitudinal studies with larger sample sizes are needed to establish associations between intracranial pressure, ocular blood flow and ONH parameters.

## 1. Introduction

Glaucoma is a multifactorial, progressive neurodegenerative disorder that may result in ONH damage, retinal ganglion cell (RGC) loss, retinal nerve fiber layer (RNFL) thinning and visual field (VF) loss [1]. Glaucoma is the most common cause of irreversible blindness worldwide, affecting more than 79 million people and predicted to reach 111.8 million by 2040 [2]. The prevalence of open-angle glaucoma rises with age, from 0.4% at ages 40–44 to 10.0% in those over 90 in Europe [3].

The vascular theory of glaucoma considers vascular dysfunction and low perfusion pressure of the ONH to play a role in the pathogenesis of the disease [4,5]. Changes in blood flow can lead to changes in the ONH. Studies have shown that patients with glaucoma have an increased eye vascular resistance index (RI) and decreased blood flow velocities, when compared to healthy individuals [6].

OCTA is a non-invasive imaging technique used to visualize the microvasculature of the retina, choroid and ONH [7,8,9]. VD, which refers to the area occupied by large vessels and microvasculature, and the flow index have both been shown to correlate with the severity of glaucoma [10,11].

Several studies have found a correlation between low ICP and glaucoma progression [6,12]. Low ICP could have a similar damaging effect on the optic nerve as increased intraocular pressure (IOP). Several experimental studies have demonstrated that a reduction in cerebrospinal fluid pressure to the ONH may lead to typical glaucomatous cupping [12,13].

The aim of this study was to evaluate the relationships between optic nerve structural, vascular, retrobulbar blood flow and ICP parameters in glaucoma patients and healthy subjects.

## 2. Materials and Methods

This prospective clinical study was performed at the Department of Ophthalmology, Hospital of the Lithuanian University of Health Sciences Kaunas Clinics, from February to August 2022. Twenty-four patients with glaucoma and twenty-five healthy subjects were enrolled in this study. The research protocol was approved by Kaunas’ regional bioethics committee (No. BE-2-115). All participants provided informed consent before participating in this study.

The inclusion criteria for the glaucoma group were the following: a confirmed clinical diagnosis of primary open-angle glaucoma (POAG), presence of changes in the ONH and VF loss consistent with glaucoma and IOP compensated with local therapy during the previous 6 months according to patients’ electronic records. Healthy subjects were age-matched volunteers with no history of glaucoma or other conditions that could bias the results. The exclusion criteria for all groups were unwillingness to participate in this study, pregnant or nursing individuals, high refractive errors (myopia and hyperopia greater than 6.0 diopters or astigmatism greater than 3.0 diopters), amblyopia, previous ocular trauma, patients with uncontrolled systemic diseases and those with a history of allergy to local anesthetics or other conditions that could bias the study results.

All patients underwent an ophthalmological exam including best-corrected visual acuity on a Snellen chart, biomicroscopy and ophthalmoscopy. A Goldmann tonometer was used to measure IOP, and a pachymeter (Alcon OcuScanRxP, Irvine, CA, USA) was used to determine central corneal thickness. Standard automated perimetry was conducted using the Humphrey 24-2 Swedish interactive thresholding algorithm perimeter (Humphrey Standard Perimetry; Carl Zeiss Meditec, 07745 Jena, Germany). VF testing was considered unreliable if fixation losses exceeded 25% or if the false-negative or false-positive errors exceeded 15%. Mean deviation (MD), pattern standard deviation (PSD) and visual field index (VFI) were assessed. An orbital Doppler device (Vittamed 205, Kaunas, Lithuania) was used for non-invasive intracranial pressure measurements in a supine position. A head frame with a fixed ultrasound transducer was placed over the closed eyelid with a small inflatable ring cuff producing pressure on the orbit and surrounding eyeball tissues. Blood flow parameters in the intracranial and extracranial segments of the OA were measured simultaneously. The value of pressure when OA blood flow signals in both intracranial and extracranial segments were equal was fixed automatically by computer system and expressed in absolute units of mmHg. The duration of the measurement procedure was up to 10 min. CDI (Mindray M7, Shenzhen, China) with a small-parts probe was used for retrobulbar blood flow measurements in the OA, CRA and SPCA. In each vessel, PSV and end-diastolic velocity (EDV) were assessed, and resistance index (RI) was calculated using Porcelot’s formula: RI = (PSV − EDV)/PSV.

**Figure 1:**
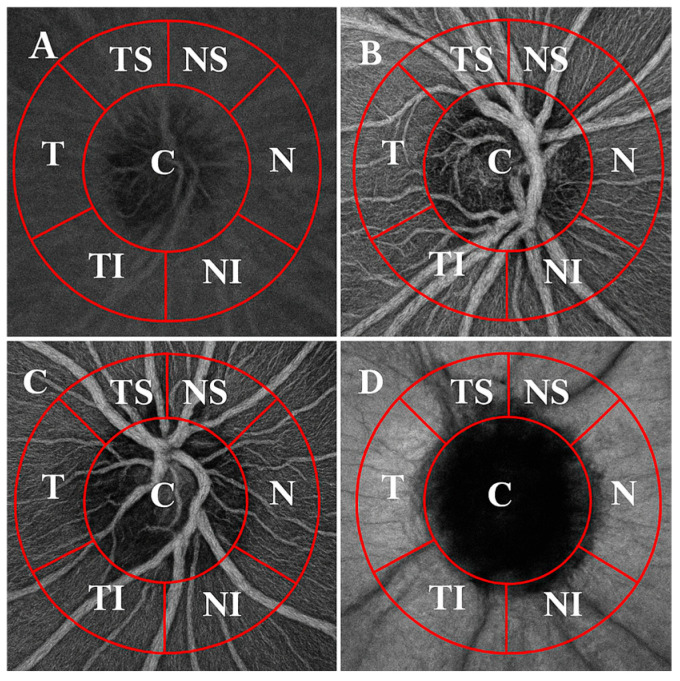
OCTA images of the ONH (3 × 3 mm) across the retinal and choroidal layers, divided into sectors. Layers: (**A**) superficial plexus, (**B**) deep plexus, (**C**) avascular retina and (**D**) choriocapillaris. Sectors: C—central; NS—superior nasal; N—nasal; NI—inferior nasal; TI—inferior temporal; T—temporal; TS—superior temporal.

VD was imaged with 3 × 3 mm scans (320 × 320 pixels) centered on the ONH. Both eyes were captured, but only one eye per patient was chosen randomly for further analysis. The OCTA images were superimposed and manually aligned with the infrared fundus image yielded by SS-OCT using ImageJ software (V.1.53k, National Institutes of Health, Bethesda, MD, USA). VD was measured across four layers (superficial capillary plexus, deep capillary plexus, avascular retina and the choriocapillaris) using a 750 µm-wide circular annulus extending outward from the outer boundary of the ONH and was divided into the following six sectors (see Figure 1): T (316–45°), TS (46–90°), NS (91–135°), N (136–225°), NI (226–270°) and TI (271–315°). VD was defined as the percentage of the area occupied by capillaries in these six sectors around the ONH. The Phansalkar threshold (radius, 15 pixels) was used to binarize the image and then “Analyse particles” was applied to count VD%. Each grid section was measured three times, and then the mean (M) value was counted to ensure accuracy.

Statistical analyses were performed using MS Excel 2010 and IBM SPSS version 25 (IBM Corporation, Armonk, NY, USA). The minimum sample size was calculated using the Altman nomogram to detect a clinically significant difference in peripapillary vessel density (VD) of the superficial plexus layer in the TI sector between glaucoma and control groups. The mean difference in VD between groups was estimated at 0.10 (10%), with an estimated standard deviation of 0.057 (5.7%), yielding a standardized effect size (Cohen’s d) of 1.75. Using a significance level of 0.05 and a power of 0.95, the required sample size was determined to be 40 subjects (20 per group). The Kolmogorov–Smirnov test was used to assess for normality of distribution, and variables were reported in terms of mean and standard deviation (SD). Paired samples *t*-test and Spearman’s correlation test were used to assess differences and correlations, with R-values indicating weak (≤0.3), medium (0.3 < r ≤ 0.75) and high (0.75 < r ≤ 1) correlations. Results with a *p*-value of less than 0.05 (*p* < 0.05) were considered to be statistically significant.

## 3. Results

A total of 49 individuals, 24 glaucoma patients and 25 healthy control subjects were included in this study. There were a few statistically significant differences in key demographic and ocular parameters found between the groups, which are represented in Table 1.

**Figure 2:**
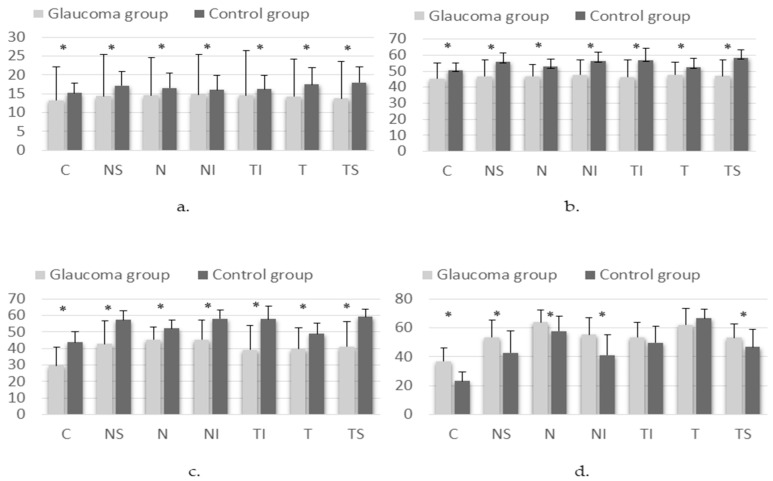
Differences in VD on OCTA scan of the ONH results in the superficial plexus (**a**), deep plexus (**b**), avascular retina (**c**) and choriocapillary layer (**d**) in glaucoma and control groups (* *p* < 0.05). Sectors: C—central; NS—superior nasal; N—nasal; NI—inferior nasal; TI—inferior temporal; T—temporal; TS—superior temporal.

ONH VDs as measured on OCTA, by layer and sector, are shown in Figure 2.

**Figure 3:**
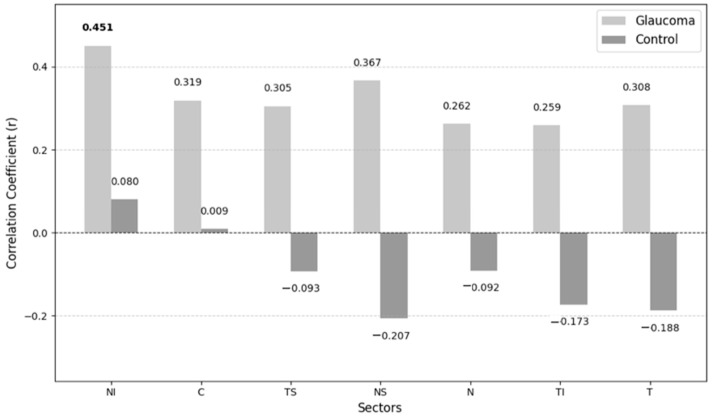
Correlations between non-invasive ICP and VD of superficial plexus layer.

**Figure 4:**
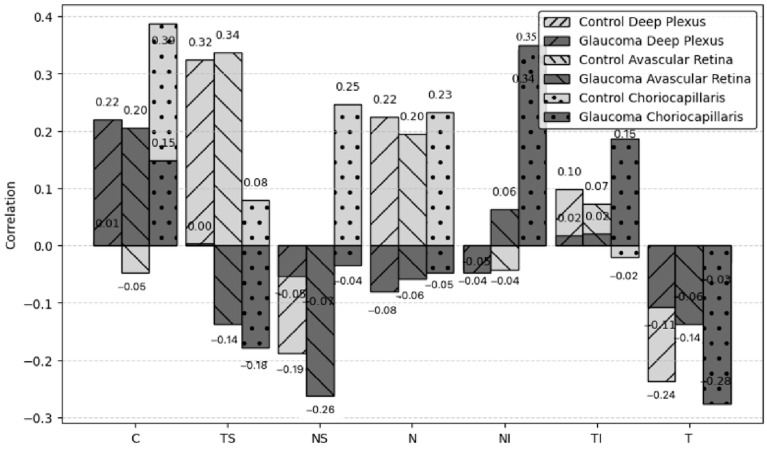
Correlations between non-invasive ICP and VD of other layers of the retina.

Correlations between the non-invasive ICP and VD of different layers in the glaucoma and the control groups are shown in Figure 3 and Figure 4.

The only statistically significant correlation observed was between non-invasive ICP and VD in the NI sector of the superficial plexus layer (*p* = 0.046). In contrast, the control group exhibits weaker correlations.

No significant correlations were observed between non-invasive ICP and VD in other retinal layers (deep plexus, avascular retina or choriocapillaris) across any sector.

**Figure 5:**
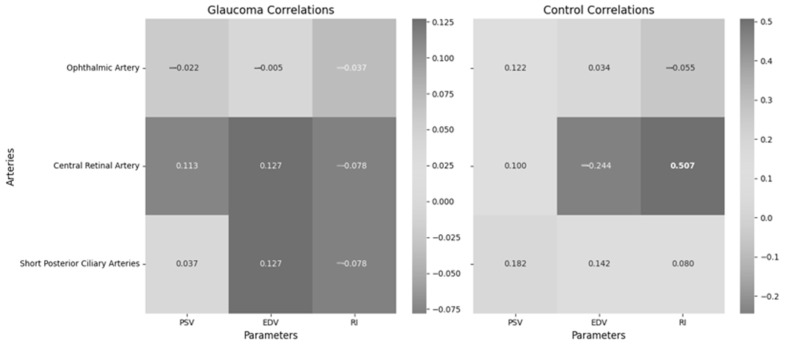
Correlations between non-invasive ICP and retrobulbar blood flow in different arteries in the glaucoma and control groups.

Figure 5 presents the correlations between non-invasive ICP and retrobulbar blood flow parameters, including PSV, EDV and RI in the OA, CRA and SPCA within the glaucoma group. These retrobulbar arteries supply critical blood flow to the optic nerve head and surrounding ocular structures, making them essential for understanding hemodynamic changes in glaucoma.

Non-invasive ICP showed no significant correlation with ophthalmic artery parameters (PSV, EDV, RI), central retinal artery parameters (PSV, EDV, RI) and short posterior ciliary artery parameters (PSV, EDV, RI) in the glaucoma group. However, non-invasive ICP showed a significant moderate correlation with central retinal artery RI in the control group.

Table 2 highlights the correlations between CRA PSV and VD across different retinal layers and sectors.

Significant moderate correlations were observed between CRA PSV and VD in various retinal layers, including the superficial plexus layer (r = 0.532, *p* = 0.008, r = 0.532, *p* = 0.008 in the temporal sector) and the avascular retina layer (r = 0.637, *p* = 0.001, r = 0.637, *p* = 0.001 in the temporal sector). In the glaucoma group, PSV and VD showed significant weak-to-moderate positive correlations, whereas the control group exhibited weak negative to weak positive correlations. This suggests a potential impairment in the autoregulatory response of glaucoma patients, which may contribute to disease progression.

## 4. Discussion

While the pathophysiology of glaucoma remains unclear, growing evidence suggests that ICP may play a significant role in this disease. In our research, we included glaucoma patients with IOP controlled with medication in order to rule out IOP effects. We found a lower mean non-invasive ICP in the glaucoma group, though the difference was not statistically significant. Loiselle et al. measured ICP non-invasively using distortion product otoacoustic emission (DPOAE) phase in 30 controls, 17 subjects with POAG and 15 with normal tension glaucoma (NTG) and found no evidence that glaucoma subjects had a reduced ICP [14]. Jonas et al. calculated ICP in 4546 subjects and found that the glaucomatous group had a significantly lower ICP than the non-glaucomatous group [15]. We found that ICP was significantly associated with the VD of the superficial plexus layer in the NI sector with no existing literature on ICP-VD correlations in glaucoma. We observed a statistically significant positive correlation between non-invasive ICP and VD in the NI sector of the superficial plexus among glaucoma patients. Conversely, the control group exhibited weaker correlations. This suggests that elevated ICP may influence ocular microcirculation in glaucoma patients, potentially contributing to disease progression. Malhotra et al. found that peripapillary capillary density was similar in controls with idiopathic intracranial hypertension, but they suggested that papilledema has differential effects on different levels of the vasculature [16]. In our study, ICP was not associated with OA, CRA and SPCA PSV, EDV or RI in the glaucoma group. Siaudvytyte et al. found lower OA blood flow velocities in NTG patients with lower ICP compared to those with higher ICP [17].

Our results showed that ONH VD in the superficial plexus, deep plexus and avascular retina was statistically significantly lower in the glaucoma group, except for the choriocapillaris layer, where ONH VD was higher. N.I. Kurysheva et al. found that the average peripapillary VD in all sectors was lower in moderate or severe POAG as compared to healthy subjects [18]. Lommatzsch et al. analyzed 80 eyes and found significantly higher VD in the NTG group in the inferior-nasal peripapillary area, at a radial peripapillary capillary layer level compared to the POAG and exfoliation glaucoma (XFG) groups and at the ONH level compared to XFG [19].

Our study identified that glaucomatous damage may be related to circulation issues in the ONH, with changes in blood flow to the OA, CRA and SPCA potentially playing a role in glaucoma development. An interesting trend observed in the glaucoma group was the presence of statistically significant weak-to-moderate positive correlations between PSV and VD. In contrast, the control group exhibited weak negative to weak positive correlations. This pattern suggests that in healthy individuals, a decrease in VD may trigger an autoregulatory response, leading to increased blood flow to compensate for reduced perfusion. However, in glaucoma patients, this compensatory mechanism appears to be impaired, potentially exacerbating disease progression by failing to adequately respond to vascular insufficiency. In related research, Koc H. et al. reported significant negative correlations between retinal RNFL thickness and OA blood flow parameters in specific glaucoma types [20]. Zegadlo A. et al. found positive correlations between RNFL thickness and OA, CRA and SPCA velocities, alongside negative correlations between RNFL thickness and the resistance index (RI) of these vessels [21]. Janulevičienė et al. similarly observed significant RNFL thinning linked with reduced retrobulbar artery velocities in open-angle glaucoma patients [22].

This study is a pilot investigation and may lack the statistical power to detect significant correlations in some retinal sectors. While some correlations were not statistically significant, we observed a noticeable trend of stronger positive correlations between ICP and VD in the POAG group compared to the control group. This trend is interesting because it suggests that ICP may play a more substantial role in the regulation of ocular blood flow in glaucoma patients, though further studies with larger sample sizes are needed to validate this finding.

The main limitation of our study is the relatively small sample size, as it was intended as a pilot study. This should be considered when interpreting the results, and future studies with larger sample sizes are needed to confirm our findings. A limitation of this study is the lack of blood pressure (BP) measurements, which could influence ICP and its correlation with ocular parameters. Future studies should consider including BP data to better assess its potential impact on ICP and ocular hemodynamics. Another limitation of this study is the lack of data on the medications used by patients, which could potentially influence ocular blood flow and ICP. Collecting detailed information on medication types and dosages would be beneficial in future studies to explore their impact on the parameters measured.

## 5. Conclusions

This study highlights a significant relationship between non-invasive intracranial pressure (ICP) and vessel density (VD) in specific sectors of the optic nerve head (ONH), particularly the inferior-nasal sector of the superficial plexus layer. Moreover, the observed reductions in ONH VD across superficial, deep and avascular retinal layers in glaucoma patients underscore the importance of microvascular health in glaucoma pathophysiology.

From a clinical perspective, these findings advocate for integrating advanced imaging modalities, such as OCTA and non-invasive ICP monitoring, into routine glaucoma assessment to better understand individual vascular and biomechanical contributions to disease progression. Further longitudinal studies with larger sample sizes are needed to elucidate causal relationships and refine patient-specific therapeutic strategies.

## Figures and Tables

**Table 1:** Demographic and ocular characteristics in glaucoma patients and healthy controls.

Parameter	Glaucoma Group (n = 24) Mean (SD)	Control Group (n = 25) Mean (SD)	*p*-Value
Age (years)	62.04 (4.72)	59.4 (5.4)	0.077
Female/Male ratio	13:11	16:9	0.485
Visual Acuity	0.91 (0.14)	0.95 (0.13)	0.301
Intraocular Pressure (mmHg)	13.83 (4.29)	14.48 (2.1)	0.507
Spherical Equivalent (D)	−0.92 (2.15)	0.68 (1.57)	0.005 ^1^
Axial Length (mm)	24.35 (2.26)	23.29 (0.94)	0.041 ^1^
Central Corneal Thickness (µm)	529.92 (36.08)	543.0 (33.47)	0.197
MD	−6.96 ± 6.2	−1.15 ± 2.28	<0.001 ^1^
PSD	6.5 ± 4.81	2.16 ± 1.49	<0.001 ^1^
VFI	81.96 ± 18.02	97.4 ± 4.74	<0.001 ^1^
Non-Invasive ICP (mmHg)	8.35 ± 2.8	8.45 ± 3.19	0.907

^1^ *p* < 0.05. VF indices—MD, PSD and VFI—showed statistically significant differences between the two groups. The glaucoma group had values indicating considerably greater visual impairment compared to the control group (*p* < 0.001).

**Table 2:** Correlations between central retinal artery PSV and VD in different retinal layers.

Layer and Sector	Parameter	Correlation (r) Glaucoma	*p*-Value Glaucoma	Correlation (r) Control	*p*-Value Control
Superficial Plexus (T)	PSV	0.532 ^1^	0.008	0.075	0.721
Deep Plexus (T)	PSV	0.469	0.021	−0.015	0.943
Avascular Retina (C)	PSV	0.413 ^1^	0.045	0.199	0.340
Avascular Retina (TS)	PSV	0.447 ^1^	0.028	0.100	0.635
Avascular Retina (NS)	PSV	0.419 ^1^	0.042	0.209	0.315
Avascular Retina (N)	PSV	0.554 ^1^	0.005	0.101	0.630
Avascular Retina (TI)	PSV	0.416 ^1^	0.043	−0.174	0.406
Avascular Retina (T)	PSV	0.637 ^1^	0.001	0.152	0.467
Choriocapillary (T)	PSV	0.467	0.021	0.152	0.470

^1^ *p* < 0.05. This table shows the Spearman’s correlation between mean PSV and mean VD for each patient in the glaucoma and control groups across various retinal layers and sectors. Marked values indicate statistically significant correlations. The correlations are calculated based on the average values of PSV and VD for each patient in their respective group.

## Data Availability

Dataset available on request from the authors.

## Abbreviations

The following abbreviations are used in this manuscript:

ICP	Intracranial pressure
SS-OCT	Swept-source optical coherence tomography
OCTA	OCT angiography
ONH	Optic nerve head
VD	Vessel density
CDI	Color Doppler imaging
OA	Ophthalmic artery
CRA	Central retinal artery
SPCA	Short posterior ciliary arteries
C	Central
NS	Superior nasal
N	Nasal
NI	Inferior nasal
TI	Inferior temporal
T	Temporal
TS	Superior temporal
PSV	Peak systolic velocity
EDV	End-diastolic velocity
RI	Resistance index
DPOAE	Distortion product otoacoustic emission
POAG	Primary open-angle glaucoma
NTG	Normal tension glaucoma
XFG	Exfoliation glaucoma
RNFL	Retinal nerve fiber layer
